# *Actinobacillus pleuropneumoniae* Possesses an Antiviral Activity against Porcine Reproductive and Respiratory Syndrome Virus

**DOI:** 10.1371/journal.pone.0098434

**Published:** 2014-05-30

**Authors:** Cynthia Lévesque, Chantale Provost, Josée Labrie, Yenney Hernandez Reyes, Jorge A. Burciaga Nava, Carl A. Gagnon, Mario Jacques

**Affiliations:** 1 Centre de recherche en infectiologie porcine et avicole (CRIPA), and Groupe de recherche sur les maladies infectieuses du porc (GREMIP), Faculté de médecine vétérinaire, Université de Montréal, St-Hyacinthe, Québec, Canada; 2 Departamento de Bioquímica, Facultad de Medicina, Universidad Juárez del Estado de Durango, Durango, México; University of Saskatchewan, Canada

## Abstract

Pigs are often colonized by more than one bacterial and/or viral species during respiratory tract infections. This phenomenon is known as the porcine respiratory disease complex (PRDC). *Actinobacillus pleuropneumoniae* (*App*) and porcine reproductive and respiratory syndrome virus (PRRSV) are pathogens that are frequently involved in PRDC. The main objective of this project was to study the *in vitro* interactions between these two pathogens and the host cells in the context of mixed infections. To fulfill this objective, PRRSV permissive cell lines such as MARC-145, SJPL, and porcine alveolar macrophages (PAM) were used. A pre-infection with PRRSV was performed at 0.5 multiplicity of infection (MOI) followed by an infection with *App* at 10 MOI. Bacterial adherence and cell death were compared. Results showed that PRRSV pre-infection did not affect bacterial adherence to the cells. PRRSV and *App* co-infection produced an additive cytotoxicity effect. Interestingly, a pre-infection of SJPL and PAM cells with *App* blocked completely PRRSV infection. Incubation of SJPL and PAM cells with an *App* cell-free culture supernatant is also sufficient to significantly block PRRSV infection. This antiviral activity is not due to LPS but rather by small molecular weight, heat-resistant *App* metabolites (<1 kDa). The antiviral activity was also observed in SJPL cells infected with swine influenza virus but to a much lower extent compared to PRRSV. More importantly, the PRRSV antiviral activity of *App* was also seen with PAM, the cells targeted by the virus *in vivo* during infection in pigs. The antiviral activity might be due, at least in part, to the production of interferon γ. The use of *in vitro* experimental models to study viral and bacterial co-infections will lead to a better understanding of the interactions between pathogens and their host cells, and could allow the development of novel prophylactic and therapeutic tools.

## Introduction

Respiratory disease in pigs is common in modern pork production worldwide and is often referred to as porcine respiratory disease complex (PRDC) [1]. PRDC is polymicrobial in nature, and occurs following infections with various combinations of primary and secondary respiratory pathogens. There are a variety of viral and bacterial pathogens commonly associated with PRDC including porcine reproductive and respiratory syndrome virus (PRRSV) and *Actinobacillus pleuropneumoniae* (*App*) [1]. Both are considered pathogens of major importance or relevance for the pig industry [1]. Furthermore, bacterial-viral co-infections can exacerbate the pathogenicity of respiratory pig diseases [1]. For example, co-infections with *Mycoplasma hyopneumoniae* and swine influenza virus (SIV) exhibited more severe clinical disease [2], PRRSV and *Streptococcus suis* co-infection experiments confirmed that PRRSV predisposes pigs to *S. suis* infection and bacteremia [3] and increases the virulence of PRRSV in pigs [4], *M. hyopneumoniae* infection increases effectiveness of PRRSV infection and lesions [5], and PRRSV infection was able to accelerate *Haemophilus parasuis* infection and loads [6]. Those studies on co-infections principally looked at the macroscopic lesions and at the clinical signs. Only a few recent studies are investigating more closely the direct interactions and mechanisms involved between the pathogens. As an example, Qiao and collaborators showed that PRRSV and bacterial endotoxin (LPS) act in synergy to amplify the inflammatory response of infected macrophages [7]. Thus, it is crucial to develop new *in vitro* models to investigate in more details the mechanistic and the interactions involved in polymicrobial infections.

Porcine reproductive and respiratory syndrome (PRRS) is the most economically devastating viral disease affecting the swine industry worldwide [8]. The etiological agent, PRRSV, possesses a RNA viral genome with ten open reading frames [8]–[10]. PRRSV virulence is multigenic and resides in both the non-structural and structural viral proteins. The molecular characteristics, biological and immunological functions of the PRRSV structural and non-structural proteins and their involvement in the virus pathogenesis were recently reviewed [8]. The disease induced by PRRSV has many clinical manifestations but the two most prevalent are severe reproductive failure in sows and gilts (characterized by late-term abortions, an increased number of stillborn, mummified and weak-born pigs) [11], [12] and respiratory problems in pigs of all ages associated with a non-specific lymphomononuclear interstitial pneumonitis [11]–[13].

*App* is the causative agent of porcine pleuropneumonia, a severe and highly contagious respiratory disease responsible for major economic losses in the swine industry worldwide [14]. The disease, transmitted by aerosol or by direct contact with infected pigs, may result in rapid death or in severe pathology characterized by hemorrhagic, fibrinous, and necrotic lung lesions. Exposure to the organism may lead to chronic infection such that animals fail to thrive; alternatively, they survive as asymptomatic carriers that transmit the disease to healthy herds. Many virulence factors of this microorganism have been well characterized [14]–[16]. To date, fifteen serotypes of *App* based on capsular antigens have been described [17], [18]. The prevalence of specific serotypes varies with geographic region [17].

Recent advances in pathogen detection methods allow better understanding of interactions between pathogens, improve characterization of their mechanisms in disease potentiation and demonstrate the importance of polymicrobial disease [1]. In the present study, the *in vitro* interactions between PRRSV and *App* in PRRSV permissive cell models were investigated. Thus, MARC-145 cells, SJPL cell line and pulmonary alveolar macrophages (PAM) were used in this study since they have been shown previously to be permissive to PRRSV infection and replication [8], [19]. Results indicate that *App* possesses a strong antiviral activity against PRRSV *in vitro.*

## Results

### PRRSV Infection Effect on *App* Bacterial Adherence

**Figure 1:**
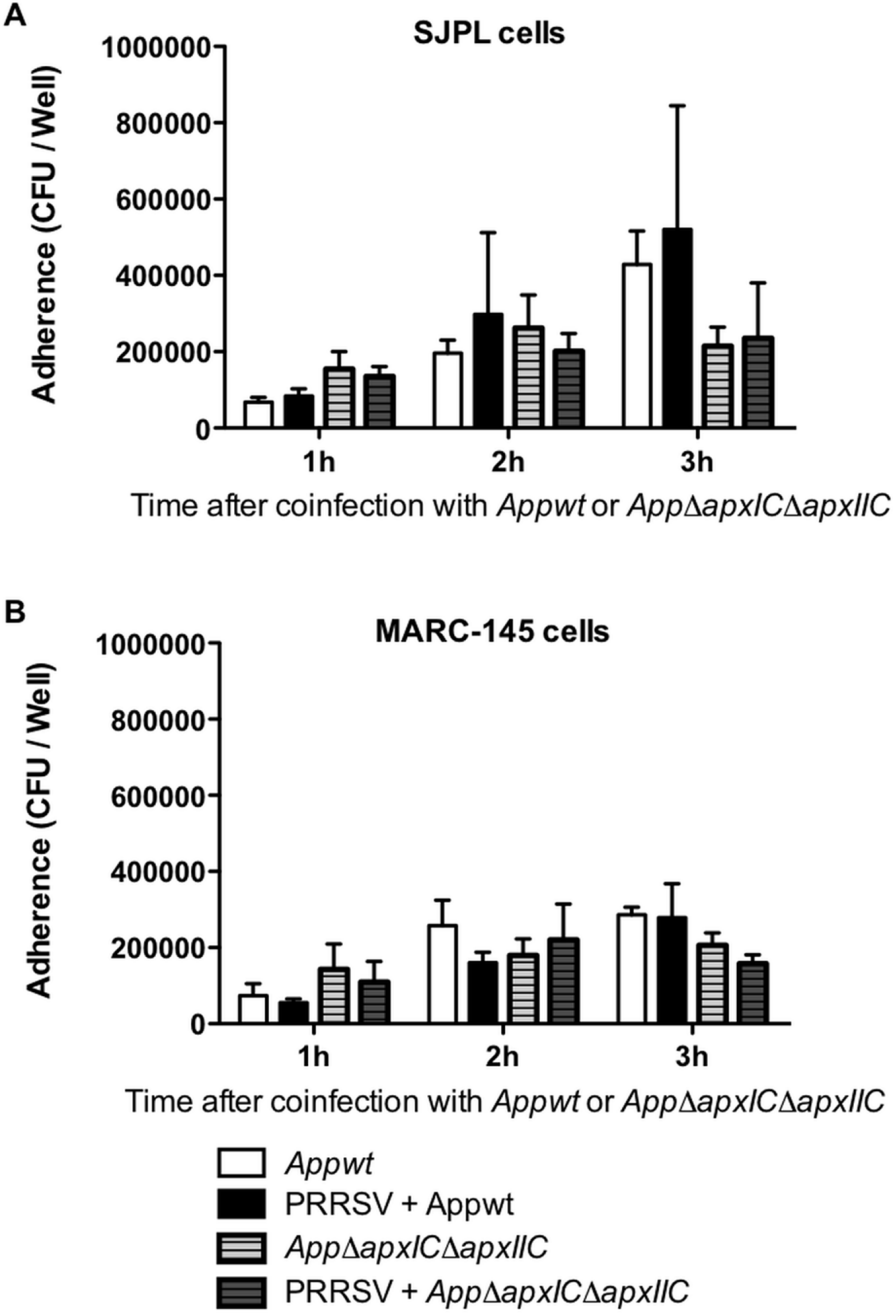
Bacterial adherence over time of *Appwt* or *AppΔapxIΔapxIIC* in PRRSV co-infected SJPL and MARC-145 cells. SJPL (A) and MARC-145 (B) cells were infected with or without PRRSV at an MOI of 0.5 during 72 hours, and then cells were co-infected with *Appwt* or *AppΔapxIΔapxIIC* at an MOI of 10. Bacterial adherence was measured in CFU per well after 1, 2 and 3 hours post bacterial infection as described in Auger *et al*., 2009 [20]. Values are presented as ± Standard Deviation (SD). No statistical significance was obtained following two-away ANOVA analysis. All experiments were repeated 3 times.

Bacterial adherence of *Appwt* and *AppΔapxIΔapxIIC* to PRRSV-infected and non-infected SJPL and MARC-145 cells was compared (Figure 1). Prior infection of both cell types with PRRSV did not significantly affect the adhesion of neither *Appwt* nor *AppΔapxIΔapxIIC* strain.

### Impact of *App* and PRRSV Co-infection on Cell Cytotoxicity

**Figure 2:**
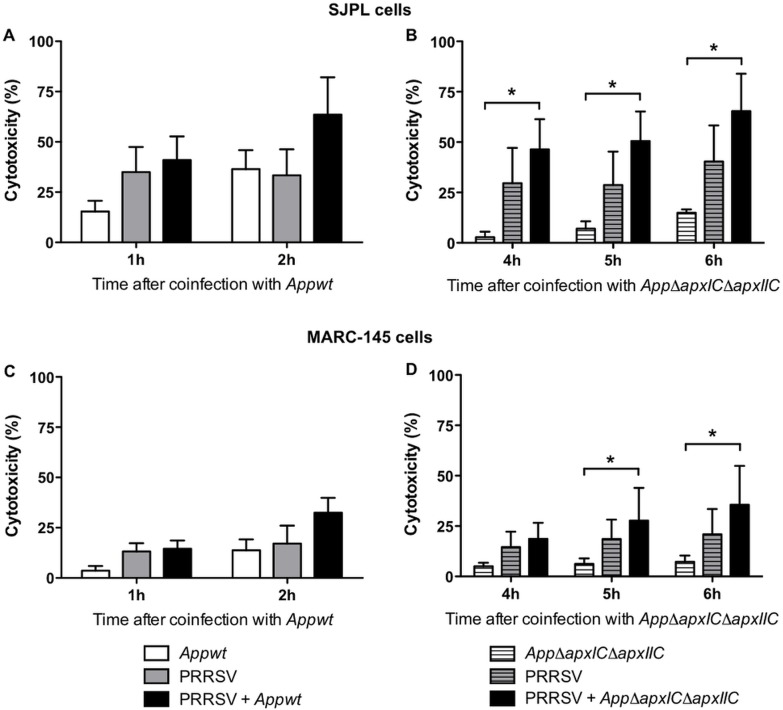
Cytotoxicity over time of *Appwt* or *AppΔapxIΔapxIIC* in PRRSV co-infected SJPL and MARC-145 cells. SJPL (A and B) and MARC-145 cells (C and D) were infected with or without PRRSV at an MOI of 0.5 during 72 hours, and then cells were co-infected with *App* (for 1 or 2 hours) (A and C, respectively) or with *AppΔapxIΔapxIIC* (for 4, 5 and 6 hours) (B and D, respectively) at an MOI of 10. Cytotoxicity was measured in % using lactate dehydrogenase (LDH) CytoTox assay [20]. Values are presented as ± Standard Deviation (SD). Two-away ANOVA analysis was used to obtain statistical data. *P<0.05. All experiments were performed 3 times.

Auger *et al*. 2009 [20] have previously published that SJPL cell death induced by *App* occurs through necrosis and not apoptosis. Consequently, based on this previous report, only a cytotoxicity experiment was performed in order to verify if PRRSV infection increases the cytotoxicity of *App*. Moreover, this assay was done to confirm that inactivation of the toxins ApxI and ApxII in the mutant *AppΔapxIΔapxIIC* reduces cell death seen with *Appwt* strain. Thus, LDH cytotoxicity assays to detect cell death were performed on cells infected with PRRSV for 72 hours and then co-infected with *Appwt* strain or *AppΔapxIΔapxIIC*. As shown in Figure 2, the cytotoxic activity of *Appwt* was higher in both cell lines after 2 hours of incubation, around 36% in SJPL cells (Figure 2A) and around 14% in MARC-145 cells (Figure 2C) compared to the one of *AppΔapxIΔapxIIC* mutant after 6 hours of incubation, which was less than 15% in SJPL cells (Figure 2B) and around 7% in MARC-145 cells (Figure 2D). As expected, the *AppΔapxIΔapxIIC* mutant is markedly less cytotoxic than the parental strain *Appwt*. Thus, *AppΔapxIΔapxIIC* mutant allows much longer incubation periods with cells and facilitate *in vitro* observation. Furthermore, co-infection with PRRSV and *AppΔapxIΔapxIIC* increased SJPL and MARC-145 cells death compared to *App* single infection (Figure 2B and D, respectively), showing an additive cytotoxicity effect of PRRSV and *AppΔapxIΔapxIIC*. Because of its markedly reduced cytotoxicity, the *AppΔapxIΔapxIIC* was used for all the subsequent experiments.

### *App* Effects on PRRSV Infection

**Figure 3:**
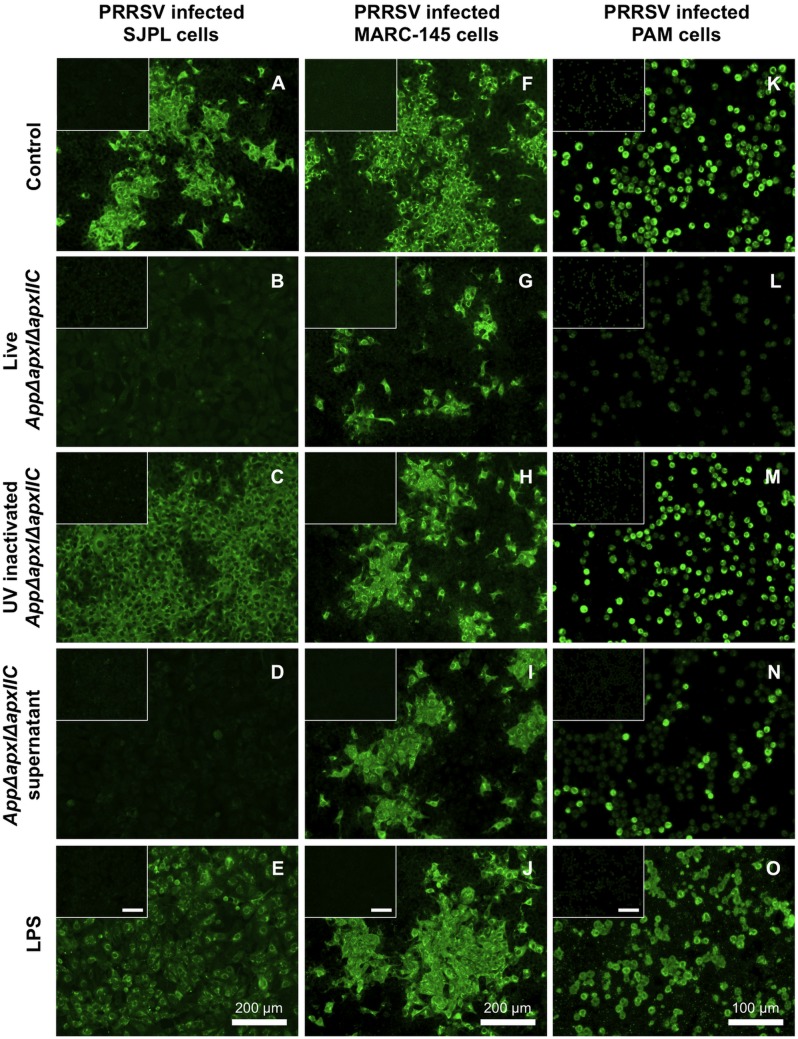
PRRSV antigen detection in SJPL, MARC-145 and PAM cells co-infected with *AppΔapxIΔapxIIC*. PRRSV N protein revealed by IFA in SJPL (A–E), MARC-145 (F–J) and PAM cells (K–O) were infected with PRRSV at an MOI of 0.5 for 4 hours (A, F and K) then co-infected with live *AppΔapxIΔapxIIC* at an MOI of 10 (B, G and L), or with UV inactivated *AppΔapxIΔapxIIC* at an MOI of 10 (C, H and M), or with *AppΔapxIΔapxIIC* supernatant (D, I and N) or treated with LPS 4 µg/ml (E, J and O) for 48 hours. Inserts are negative control where cells were not infected with PRRSV. White scale bar represents 200 µm for SJPL and MARC-145 cells, and 100 µm for PAM cells. Pictures were taken at 100X magnification for SJPL and MARC-145 cells, and 200X for PAM cells.

In SJPL cells, co-infection with *AppΔapxIΔapxIIC* and PRRSV shows absence of PRRSV N viral protein detection by IFA compared to control where SJPL cells were infected with PRRSV alone (Figure 3A) suggesting an inhibition of PRRSV infection and/or replication (Figure 3B). MARC-145 cell line was used to compare results obtained with SJPL cell line since MARC-145 cells are the most common cells used during *in vitro* PRRSV studies. Interestingly, results were different between the two cell lines. In PRRSV infected MARC-145 cells, only a small reduction of cells expressing the PRRSV N protein was observed following a co-infection with *AppΔapxIΔapxIIC* (Figure 3G). Thus, SJPL cells were qualitatively more responsive to the *App* antiviral affect than MARC-145 cells. Moreover, since SJPL cells were recently shown to be from monkey origin [21] and not from swine as first described [22], evaluation of the antiviral activity of *App* was tested in a porcine relevant cell model, the PAM cells. Co-infection with *AppΔapxIΔapxIIC* and PRRSV in PAM cells also presented total absence of PRRSV N protein detection (Figure 3L), as in SJPL cells (Figure 3B), suggesting that *AppΔapxIΔapxIIC* can also inhibits PRRSV in PRRSV’s *in vivo* porcine target cells, the porcine alveolar macrophages. Incubation with UV-inactivated *AppΔapxIΔapxIIC* bacteria after PRRSV infection allowed the detection of N proteins of PRRSV by IFA in all cell types (Figure 3C, 3H and 3M) showing that UV-inactivated bacteria were not able to block PRRSV infection. Interestingly, the bacteria-free culture supernatant of *AppΔapxIΔapxIIC* also effectively blocked PRRSV infection in SJPL and PAM cells (Figure 3D and 3N, respectively). A weak inhibition was observed in MARC-145 cells (Figure 3I). pH did not vary between all the tested conditions, being stable at around 7.3±0.1. The active metabolites present in the culture supernatant did not seem to be *App* LPS (Figure 3E, 3J and 3O) nor peptidoglycan fragments (assayed with NOD1 or NOD2 ligands) (Figure S1D and S1F, respectively). Dilutions of *AppΔapxIΔapxIIC* supernatant showed a dose-dependent effect on PRRSV’s detection by IFA. A 1∶2 dilution resulted in twice as much PRRSV N protein when observed with IFA (data not shown). The loss of antiviral activity of *AppΔapxIΔapxIIC* supernatant was observed with 1∶10, 1∶20 and 1∶40 dilutions.

**Figure 4:**
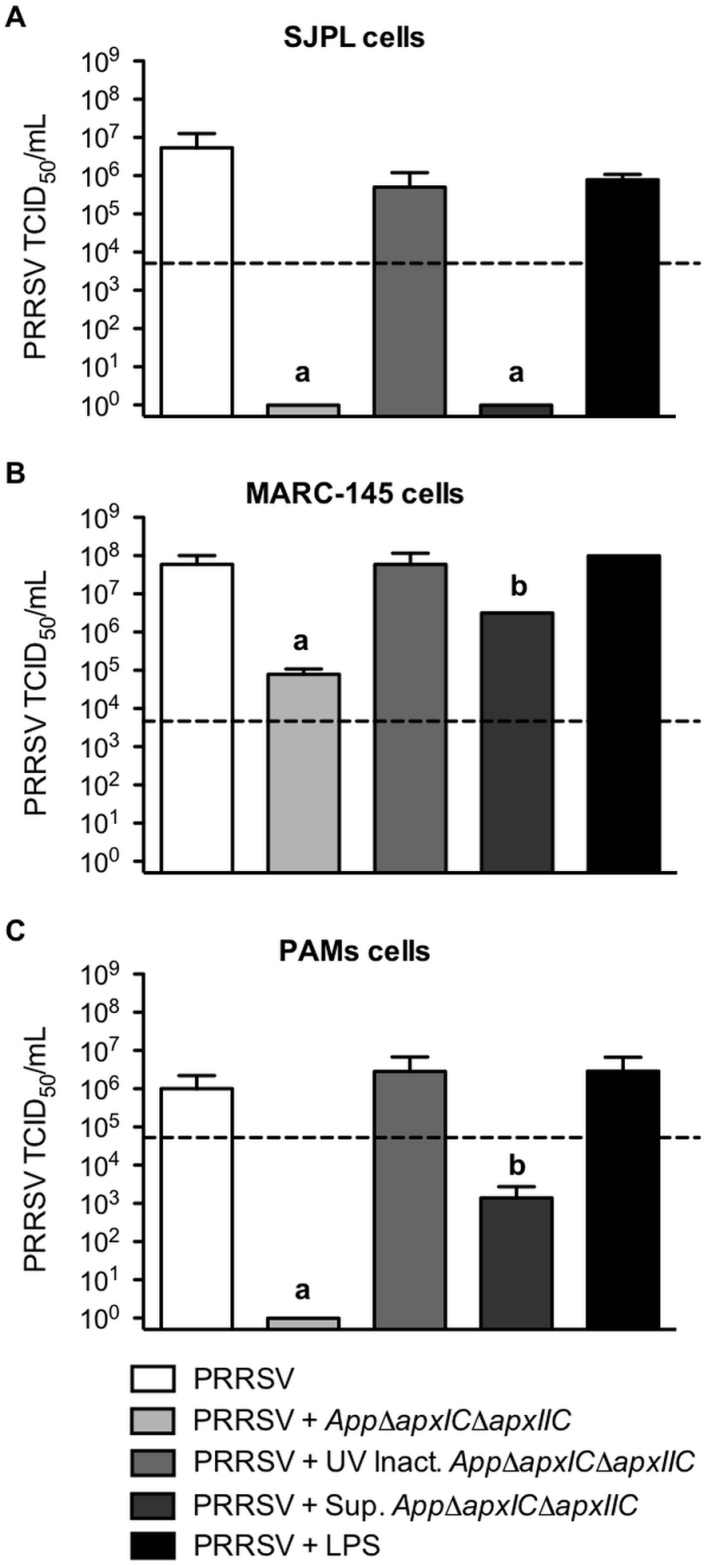
PRRSV titer in *App* treated SJPL, MARC-145 and PAM cells. SJPL (A), MARC-145 (B) and PAM (C) cells were infected with PRRSV MOI of 0.5 for 4 hours and then co-infected with *AppΔapxIΔapxIIC* MOI of 10, or with UV inactivated *AppΔapxIΔapxIIC* MOI of 10, or treated with LPS (4 µg/ml) or culture supernatant of *AppΔapxIΔapxIIC* for 48 hours. PRRSV titer was determined on MARC-145 cells by the Kärber method. Values are presented as ± Standard Deviation (SD). One-away ANOVA analysis was used to obtain statistical data. When bars within a cell type are labeled with superscripts letters, it indicates that these sets of data are statistically different from the other bars (P<0.05).

PRRSV titers were measured to confirm IFA observations and to quantify the inhibitory effect of *AppΔapxIΔapxIIC* on PRRSV infection. SJPL, MARC-145 and PAM cells were infected or treated as described previously. In SJPL cells after 72 hours post PRRSV infection, viral titer obtained was 6.25 log10 TCID_50_/ml (Figure 4A), in MARC-145 cells, was 7.6 log10 TCID_50_/ml (Figure 4B) and in PAM cells, 6.0 log10 TCID_50_/ml (Figure 4C). Co-infection with *AppΔΔapxIΔapxIIC* or treatment with its culture supernatant blocked completely PRRSV replication (*P*<0.01) in SJPL cells (Figure 4A). But in MARC-145 cells, their antiviral effect on PRRSV replication was markedly less efficient. More specifically, in MARC-145 cells, PRRSV titers were 4.9 log10 TCID_50_/ml (which correspond to a 751 fold decrease compared to PRRSV non-treated infected cell) and 6.5 log10 TCID_50_/ml (which correspond to a 19 fold decrease compared to PRRSV non-treated infected cell) for *AppΔapxIΔapxIIC* (*P*<0.01) and its cell-free culture supernatant (*P*<0.05) treated cells, respectively (Figure 4B). In PAM cells, results obtained with PRRSV’s titration showed that live *AppΔapxIΔapxIIC* completely blocked PRRSV replication (*P*<0.001) and that its culture supernatant significantly inhibits PRRSV infection in PAM, reducing its amount of infectious virions to 2.9 log10 TCID_50_/ml (*P*<0.001 compared to PRRSV infection at 10^6^ TCID50/mL) which correspond to a 1250 fold decrease (Figure 4C). Stimulation of the cells with *App* purified LPS or co-infection with UV inactivated bacteria did not have any effect on PRRSV titer in all cell types (Figure 4A, 4B and 4C). Those results confirm the IFA data obtained previously. In addition, it is important to note that inhibition in PAM is total with live *AppΔapxIΔapxIIC* as observed previously in SJPL cells and below PRRSV inoculum when treated with *AppΔapxIΔapxIIC* cell culture supernatant. Thus, those results indicate that *AppΔapxIΔapxIIC* antiviral effect against PRRSV can be observed not only in SJPL cells but also in porcine alveolar macrophages.

### Fractionation of Cell Culture Supernatant of *AppΔapxIΔapxIIC*

**Figure 5:**
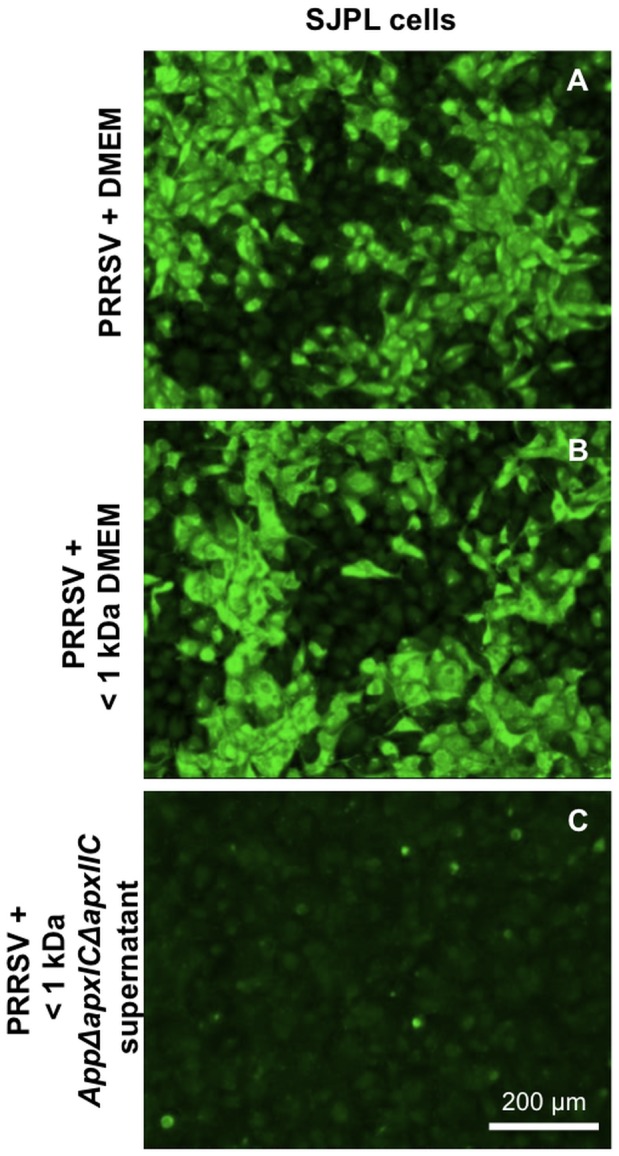
*AppΔapxIΔapxIIC* cell culture supernatant <1 kDa fraction antiviral activity against PRRSV. Detection of the N viral protein in PRRSV infected SJPL cells by immunofluorescence. SJPL cells were infected with 0.5 MOI of PRRSV for 4 hours then incubated with DMEM culture medium alone (DMEM) (A) or either a DMEM culture medium fraction of <1 kDa (DMEM <1 kDa) (B) or a *AppΔapxIΔapxIIC* cell culture supernatant <1 kDa fraction (*App*<1 kDa) (C) added to complete SJPL culture medium for 48 hours. White scale bar represents 200 µm. Pictures were taken at 100X magnification.

Fractionation of the cell culture supernatant of *AppΔapxIΔapxIIC* indicated that the ihnibitory effect on PRRSV infection is mediated by small *App* metabolite(s) weighting <1 kDa (Figure 5C). The same results were obtained with all small fractions tested, <3 (Figure S2D), 10 (data not shown) and 50 kDa (Figure S2F). Additionally, treatment at 56°C for 30 min of these low molecular weight *App* metabolite(s) did not inactivate their ihnibitory effect on PRRSV infection and/or replication in SJPL cells, showing that those *App* antiviral metabolites are heat-resistant (data not shown).

### Antiviral Efficacy of *AppΔapxIΔapxIIC* Cell Culture Supernatant against Several other Viruses

Since *AppΔapxIΔapxIIC* cell culture supernatant inhibits PRRSV replication, other viruses were tested in order to verify if this inhibition is virus specific or if it is a general antiviral effect. First, the SJPL cells permissivity was tested in regards to different DNA genome viruses such as: BAV3, BHV-1, BHV-4, CPV, EHV-1, and PCV2; as well as RNA genome viruses such as: BVDV-1, Influenza H1N1, and Influenza H3N2. BAV3, BHV-1, EHV-1, BVDV-1, Influenza H1N1, and Influenza H3N2 viruses were able to infect and replicate in SJPL cells (Table 1). Thus, treatment with *AppΔapxIΔapxIIC* culture supernatant was performed after infection with those viruses in SJPL cells, to verify its spectrum of antiviral activity. Overall, 50% of the viruses tested that are able to replicate in SJPL cells (excluding PRRSV) were inhibited by *AppΔapxIΔapxIIC* cell culture supernatant. Those inhibited viruses were: EHV-1, Influenza H1N1 and H3N2. However, it is important to note that the inhibition of PRRSV replication observed following treatment with *AppΔapxICΔapxIIC* supernatant was significantly higher compared to than the inhibition observed against EHV-1, Influenza H1N1 and H3N2 (Table 1). These results are important because they indicate that SJPL cells were still able to allow the replication of several viruses in the presence of *AppΔapxIΔapxIIC* cell culture supernatant, indicating that the SJPL cells are still metabolically active and fit for viruses’ replication.

**Table 1:** Antiviral activity of *AppΔapxIΔapxIIC* supernatant against several animal DNA and RNA viruses in SJPL infected cells.

Viruses	Virus titer		Relative virus replication inhibition
	Without *AppΔapxIΔapxIIC*	With *AppΔapxIΔapxIIC*	
	(TCID_50_ log10 ± SD)		
**DNA genome**			
BHV-4	Neg	-	-
CPV	Neg	-	-
PCV2	Neg	-	-
BAV3	2.75±0.35	2.88±0.18	0.74±2.45
BHV-1	4.54±0.48	4.42±0.59	1.32±5.75
EHV-1	5.00±0.71	3.75±0.35 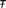	17.78±6.17
**RNA genome**			
BVDV-1	4.38±0.18	4.25±0.35	1.35±2.45
H1N1	5.40±0.57	4.23±0.50*	14.8±5.75
H3N2	4.85±0.50	3.82±0.45**	10.72±4.68
PRRSV	5.44±0.56	1.61±0.59***	6760.83±6.46*^a^*

All experiences were performed at least 2 times.

Statistical *P* value compared to *AppΔapxIΔapxIIC* untreated cells: 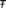 *P* = 0.15.

Statistically significative compared to *AppΔapxIΔapxIIC* untreated cells: *P<0.05, **P<0.01, ***P<0.001.

Statistically significative compared to other viruses: *^a^P*<0.01.

### Effect of *AppΔapxIΔapxIIC* Cell Culture Supernatant on the Mrna Level of Type I and Type II IFNs

**Figure 6:**
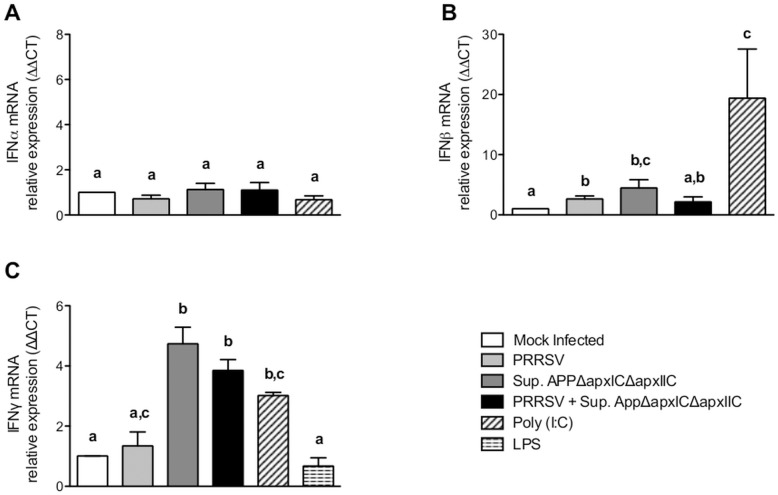
*AppΔapxIΔapxIIC* cell culture supernatant and PRRSV effects on mRNA quantification of type I (IFNα, IFNβ) and type II (IFNγ) interferons. qRT-PCR results expressed in relative expression (ΔΔCT) for IFNα (A), IFNβ (B) and IFNγ (C) in SJPL cells. The cells were mock infected or infected with 0.5 MOI of PRRSV for 4 hours then treated without or with *AppΔapxICΔapxIIC* cell culture supernatant for 48 hours. Poly (I:C) and LPS were used as positive controls. Data labeled with superscripts of different letters indicates that these sets of data are statistically different (P<0.05).

Since the levels of mRNA expression of type I (IFNα and IFNβ) and type II (IFNγ) interferons are known to be implicated in the cellular antiviral effect against PRRSV [23]–[26], mRNA levels of those cytokines were measured by qRT-PCR (Figure 6). No modulation of IFNα was observed in any of the tested conditions, including the Poly I:C control. This observation was also previously made by Provost *et al*., 2012 [19]. PRRSV infection in SJPL cells significantly increased IFNβ levels compared to mock infected cells, as previously described in Provost *et al*., 2012 [19]. Treatment with *AppΔapxICΔapxIIC* supernantant alone induced a significant increase of IFNβ mRNA compared to mock infected cells, but co-treated cells did not showed a significant increase compared to mock infected cells. PRRSV infection in SJPL cells did not modulate IFNγ mRNA levels. However, treatment with *AppΔapxICΔapxIIC* supernantant alone or as co-treatment significantly increased IFNγ mRNA compared to mock infected SJPL cells.

## Discussion

Many studies have previously shown that respiratory viral infections can increase bacterial adherence to cells. For example, influenza A infection increases adherence of *Streptococcus pyogenes* to MDCK cells [27], rhinovirus infection increases adherence of *Streptococcus pneumoniae* to cultured human airway epithelial cells [28], and respiratory syncytial virus (RSV), human parainfluenza virus 3 (HPIV-3), and influenza virus increase the adherence of *Haemophilus influenzae* and *S. pneumoniae* to respiratory epithelial cells [29]. However, in the present study, no modulation of *App* adherence was observed when cells were infected with PRRSV.

*Appwt* induced, as expected, a high percentage of cytotoxicity in SJPL cells (Figure 2). Its derivative, *AppΔapxIΔapxIIC*, that is expressing the non-activated toxins ApxI and ApxII, showed a much lower cytotoxicity in SJPL cells. Furthermore, as previously described in Provost et al. 2012, PRRSV infection in SJPL cells induced a significant increase of cell death [19]. However, co-infection with PRRSV and *AppΔapxIΔapxIIC* did not result in a significant increase of cell death when compared to PRRSV infection alone, supporting that *AppΔapxIΔapxIIC* is less (if not) toxic to eukaryotic cells and that cytotoxicity is mainly caused by PRRSV in co-infected cells. Interestingly, this less toxic *App* mutant enables longer exposure in *in vitro* experiments and allowed us to observe *App*’s antiviral effect on PRRSV.

The antiviral effect of *AppΔapxIΔapxIIC* was first observed on SJPL cells co-infected with PRRSV (Figure 3). Subsequently, other results showed that the antiviral activity was also present in the bacterial supernatant and was not due to *App* purified LPS, nor NOD ligands, but probably to low molecular weight metabolites of <1 kDa. Inhibition of PRRSV replication by *AppΔapxIΔapxIIC* is not generated by contact between bacterial and eukaryotic host cell, since it was also observed with *App* cell culture supernatant; thus without the presence of *App* bacterial cells. Furthermore, this antiviral effect is not only observed in SJPL cells but also in the PRRSV natural host target cells, i.e. PAM. This suggests that the antiviral action of *AppΔapxIΔapxIIC* can be efficient in different cell species and types. Viral inhibition in PAM cells was complete in presence of the bacteria *AppΔapxIΔapxIIC* and was partial when treated with its cell culture supernatant. Other combinations of treatments have been tested. Data obtained gave some information about the mechanism of the antiviral activity of *AppΔapxIΔapxIIC* supernatant. Overall, they suggested that *AppΔapxIΔapxIIC* supernatant’s antiviral activity is not interfering with PRRSV attachment and entry. Other experiments are currently in progress to further investigate by which mechanisms the *AppΔapxIΔapxIIC* supernatant is inhibiting PRRSV replication.

Despite the fact that MARC-145 and SJPL are of monkey origin, they are phenotypically distinct as demonstrated by our group in Provost *et al.* (2012) [19]. In this previous report, we demonstrated that SJPL and MARC-145 cells do not have the same division rate and that the development of the cytopathic effect (CPE) induced by PRRSV in SJPL cells was delayed compared to MARC-145 cells. Furthermore, the cytokine profiles after PRRSV infection were different between the two cell lines. These results suggested that PRRSV infection could be different in each. Thus, the difference in PRRSV infection between both cell lines could explain the difference observed for the *AppΔapxIΔapxIIC* supernatant antiviral activity.

Type I IFNs, produced by many cell types, are part of the innate immunity response [30]. Moreover, it is well known in the literature that type I IFNs are often part of the cellular response against viral infections, including PRRSV infections [23], [25]. Results of this study showed that there is no modulation of IFNα mRNA levels. IFNβ mRNA levels were increased in PRRSV and in *AppΔapxIΔapxIIC* supernatant alone but no significant increase was observed in the PRRSV + *AppΔapxIΔapxIIC* supernatant condition when compared to mock infected cells. Thus, the impaired IFNβ expression following co-treatment might be due to PRRSV replication which might block IFN production induced by *AppΔapxIΔapxIIC* supernatant. Additionally, those results demonstrate that since PRRSV can inhibit type I IFN induction and signalling [31]–[34], antiviral activity induced by *AppΔapxIΔapxIIC* supernatant may not rely on its ability to induce IFNβ. However, this does not mean that IFNβ is not part of the antiviral activity of *AppΔapxIΔapxIIC* supernatant, since most viruses are still sensitive to type I IFNs.

Type II IFNγ, mainly produced by activated T cells and Natural Killer cells, is mostly responsible for adaptive Th1 response, which is part of cell-mediated immunity [35]. Furthermore, its implication in antiviral response against PRRSV was also demonstrated [24], [26]. Nonetheless, IFNγ mRNA levels in SJPL cells were significantly increased by *AppΔapxIΔapxIIC* supernatant alone and in PRRSV + *AppΔapxIΔapxIIC* supernatant condition. This observation might give a clue by which cellular response *AppΔapxIΔapxIIC* supernatant induces its antiviral effect; i.e. via the increased of IFNγ mRNA levels by the cell. However, it is important to mention that it is not known if SJPL cells possess IFNγ receptors, which are necessary for IFNγ mediated signalling. Further investigations are needed to confirm this hypothesis.

PRRSV can lead to persistent infections [36], [37] and current PRRSV vaccines are not yet optimal, since they lack the ability to induce a strong immune response and since they do not provide complete immunity against homologous PRRSV infections (for review see [38], [39]). Moreover, most PRRSV vaccines are live attenuated virus and thus present a safety issue; some vaccinated pigs were shown to produce shedding of virulent PRRSV particles [40]. Thus, it is important to further investigate new possible ways to control PRRSV infections. In that regards, an antiviral molecule or metabolite might be a good alternative to the currently used vaccines. Recently published studies showed few compounds that can inhibits PRRSV as glycosides, terpenoids, coumarins, isoflavones, peptolides, alkaloids, flavones, macrolides [41], N-acetylpenicillamine [42], cyclosporine A [43], sodium tanshinone IIA sulfonate [44], flavaspidic acid AB [45], Ribavirin [46], and morpholino oligomer [47], or compounds derived from plant as a polysaccharide isolated from *Achyranthes bidentata* [48] or a mushroom extract from *Cryptoporus volvatus* [49]. However, there is no commercially available antiviral drug against PRRSV on the market.

In conclusion, to the best of our knowledge, this is the first description of an *App* antiviral activity. This study might lead to the development of a new treatment against PRRSV derived from *App* cell culture supernatant. However, more investigations are needed to identify and/or purify the target metabolite(s) secreted by *App* before generating a possible new antiviral molecule against PRRSV. Moreover, since we have demonstrated that the antiviral effect of the metabolite(s) secreted from *App* is not only specific to PRRSV, but also effective against other RNA viruses, this antiviral activity might as well lead to a new antiviral treatment. For example, molecules such as Ribavirin, which is currently used against human respiratory syncytial virus (RSV) [50], [51] and hepatitis C infection [52], was initially demonstrated to have a broad antiviral activity against animal viruses [53]. This study might therefore allow the development of a new antiviral molecule against PRRSV, but also against other viruses such as influenza.

## Materials and Methods

### Cells

All cells products were ordered from Invitrogen Corporation GibcoBRL (Burlington, ON, CA) unless specified. MARC-145 cells, a subclone of African green monkey kidney MA104 cells, were grown in minimum essential medium (MEM) supplemented with 10% of foetal bovine serum (FBS) (Wisent Inc, St-Bruno, QC, Canada), 0.1 mM HEPES, 2 mM L-glutamine, 10 U/mL of penicillin, 10 µg/mL of streptomycin and 250 g/L antibiotic-antimitotic solution [54]. The SJPL cell line (St. Jude porcine lung epithelial cell) was provided by Dr. R.G. Webster (St. Jude Children’s Hospital, Memphis, TN, USA) [22] and later was demonstrated to be from monkey origin [21]. This cell line was grown in Dulbecco’s modified Eagle’s medium (DMEM) supplemented with 10% FBS (Wisent Inc), 1 mM sodium pyruvate, 2 mM L-glutamine, 1 µM MEM nonessential amino acid, 10 U/mL of penicillin, 10 µg/mL of streptomycin and 250 g/L antibiotic-antimitotic solution and, 100 mg/L gentamicin. Porcine alveolar macrophages (PAM) were harvested from lungs of 2 to 14 weeks old pigs as described previously [19]. Pigs were sacrificed following ethic protocol 12-Rech-1640 approved by our institutional ethic committee (Comité d’éthique de l’utilisation des animaux – CÉUA) following the guidelines of the Canadian Council on Animal Care. Briefly, an instillation of the lungs with PBS containing 10 units/mL penicillin, 10 µg/mL streptomycin and 100 mg/L gentamicin was realized. Then, phosphate buffer saline solution (PBS) was collected and PAM removed following low speed centrifugation. Cells were washed with DMEM medium complemented with 2 mM L-glutamine, 0,1 mM HEPES, 1 µM non-essential amino acids, 250 g/L amphotericin B (Wisent Inc), 10 units/mL penicillin, 10 µg/mL streptomycin and 100 mg/L gentamicin. Cells were then collected following low speed centrifugation and were resuspended in freezing medium (same as wash medium plus 20% foetal bovine serum and 10% DMSO (Sigma-Aldrich, St-Louis, MO, USA)) and slowly frozen, than stored in liquid nitrogen until further utilization. PAM cells were cultured for 24 hours in complete DMEM prior to assay. All cells were cultured and infected at 37°C in 5% CO_2_ atmosphere.

### Bacterial and Viral Strains

The *App* strains used in this study were the S4074 serotype 1 reference wild type strain (*App*wt) and a mutant of this strain (MBHPP147) producing non-active ApxI and ApxII toxins (*AppΔapxICΔapxIIC)*, kindly provided by Ruud P.A.M. Segers (MSD Animal Health, Boxmeer, The Netherlands). *App* strains were cultured on brain heart infusion (BHI) broth and/or agar (Gibco) supplemented with 15 µg/ml nicotinamide adenine dinucleotide (NAD) at 37°C in 5% CO_2_. The PRRSV strain used in this study was the Canadian genotype II reference strain IAF-Klop [55].

### Adherence Assay

For the adherence assay, 10^5^ epithelial cells/well were seeded into 24 well-tissue culture plates (Sarstedt, Numbrecht, Germany) and incubated overnight (O/N). Cells were infected with PRRSV at 0.5 multiplicity of infection (MOI; virus particles or bacterial cells per cell). *App*wt and *AppΔapxIΔapxIIC* from an overnight culture grown at an OD_600 nm_ of 0.6 were resuspended in complete cell culture medium to a concentration of 10^6^ CFU/ml. One ml of either suspension was added to each well at an MOI of 10 after 72 hours PRRSV infection, and plates were incubated for 1, 2 or 3 hours. Non-adherent bacteria were removed by washing four times with Dulbecco's Phosphate-Buffered Saline (DPBS) (Gibco). Cells with adherent bacteria were released from the wells by adding 100 µl of 1X trypsin-EDTA (Gibco) and resuspended in 900 µl DPBS buffer. Serial dilutions were performed and poured on agar plates to determine the number of bacteria that adhered to the epithelial cells. Bacteria colonies were counted as colonies forming unit per well (CFU/well) as described by Auger *et al*., 2009 [20].

### Cytotoxicity Detection Assay

For the cytotoxicity detection assay, 10^5^ epithelial cells/wells were seeded into 24 well-tissue culture plates (Sarstedt) and incubated O/N. Cells were infected with PRRSV at 0.5 MOI. *Appwt* and *AppΔapxIΔapxIIC* from an overnight culture grown at an OD_600 nm_ of 0.6 were resuspended in complete cell culture medium to a concentration of 10^6^ CFU/ml. One ml of either suspension was added to each well at an MOI of 10 after 72 hours PRRSV infection, and plates were incubated for 1 or 2 hours with *Appwt* or for 4, 5 and 6 hours with *AppΔapxIΔapxIIC.* The cellular cytotoxicity was determined using the lactate dehydrogenase (LDH)-measuring CytoTox 96 nonradioactive cytotoxicity assay (Promega, Madison, WI) as described by the manufacturer. Noninfected cells were used as a negative control, while total cell lysate was used for the 100%-cytotoxicity positive control, since all LDH is released when cells are mechanically lysed. Optical densities were measured at 490 nm with a Power Wave X340 (Biotek Instruments Inc, Winooski, VT) microplate reader and used to calculate the percentage of cytotoxicity [55].

### Immunofluorescence Assay

The presence of PRRSV antigens in infected cells was determined by an immunofluorescence assay (IFA). Cells were infected or treated as described below. Following treatment and/or infections, cells were fixed with a 4% paraformaldehyde (PFA) solution prepared as previously described [19]. Mock-infected or non-treated cells were used as negative controls. After an incubation period of 20 minutes at room temperature, the PFA solution was removed and cells were washed three times with Phosphate buffer solution (PBS). Then, cells were incubated during 10 minutes at room temperature with a PBS solution containing 0.1% Triton X-100 for cell membrane permeabilization. After removing the Triton X-100 solution, cells were washed three times with a PBS-Tween 20 solution (PBS containing 0.02% Tween 20). Thereafter, cells were incubated 30 minutes with PBS containing 0.02% Tween 20 and 1% foetal bovine serum albumin. Then, the α7 rabbit monospecific antisera (anti-N PRRSV protein) [55] was diluted 1/100 in the blocking solution and added to the cells and incubated at 37°C for 90 minutes. Cells were then washed and incubated for 60 minutes with the blocking solution containing a 1/160 dilution of anti-rabbit specific antiserum FITC conjugated (Sigma). Finally, cells were visualized using a DMI 4000B reverse fluorescence microscope, image of the cells were taken with a DFC 490 digital camera and the images were analyzed using the Leica Application Suite Software, version 2.4.0 (Leica Microsystems Inc., Richmond Hill, Canada).

### Antiviral Activity of *AppΔapxIΔapxIIC* Against PRRSV

Cells were infected with 0.5 MOI of PRRSV and incubated in DMEM without serum or other additives for 4 hours, then all non-attached virus were removed from the medium with soft washing step using PBS. Thereafter fresh medium was added. *AppΔapxIΔapxIIC* from an overnight culture grown at an OD_600 nm_ of 0.6 were resuspended at an MOI of 10 in complete cell culture medium to a concentration of 10^6^ CFU/ml. To obtain *AppΔapxIΔapxIIC* UV inactivated, resuspended *AppΔapxIΔapxIIC* at an MOI 100∶1 were inactivated for 2 hours under UV light (315 nm) in a rocking petri dish and their inactivation was confirmed by plating on BHI-NAD. To obtain *AppΔapxIΔapxIIC* supernatant, resuspended *AppΔapxIΔapxIIC* at an MOI of 10 were centrifuged at 500 g for 15 minutes and harvested supernatants were passed through a 0.22 µm filter to remove all residual bacteria. Bacterial culture supernatants were further fractionated through ultrafiltration membranes with cut-off of 50, 10, 3 (Amicon Ultra-15, Millipore, Billerica, MA) or 1 kDa (Macrosep 1K, Pall Life Sciences, Port Washington, NY) to obtain *AppΔapxIΔapxIIC* cell culture supernatant fractions. *AppΔapxIΔapxIIC* supernatant was also diluted 1∶2, 1∶10, 1∶20, 1∶40. One ml of the suspensions was added to each well 4 hours after PRRSV infection, and plates were incubated for 48 hours. pH measurements were performed directly in the wells of treated SJPL cells using an Accumet basic AB15 pH meter (Fisher Scientific, Ottawa, ON). The presence of PRRSV N antigen was determined by IFA. The infectious dose of the virus was determined from serial dilutions and calculated by the Kärber method [56]. Briefly, samples infected by PRRSV were subjected to three cycles of freeze-thaw and cellular suspensions were then clarified by low speed centrifugation at 1200 g for 10 minutes. Supernatants were serially diluted then used to infect MARC-145 cells in a 96-well tissue culture plate. The plate was incubated for 96 hours. Virus titers were expressed in tissue culture infectious dose 50 per ml (TCID_50_/ml). Presence of PRRSV was also evaluated by qRT-PCR using a commercial kit (Tetracore Inc., Rockville, MD, USA) as previously described [57].

### Treatment with LPS and NOD Ligands

Cells were infected with PRRSV at 0.5 MOI of in DMEM without serum and other additives and incubated for 4 hours. Then infected cells were washed and fresh medium was added. Cells were treated with 4 µg/ml of LPS purified from *Appwt* [58], or 100 to 1,000 ng/ml of C12-iE-DAP (a NOD1 ligand, InvivoGen, San Diego CA), or 100 to 1,000 ng/ml of L18-MDP (a NOD2 ligand, InvivoGen) for 48 hours. The presence of PRRSV N protein was determined by IFA. The virus titer was determined as described above.

### *App* Cell Culture Supernatant Antiviral Activity Against other DNA and RNA Viruses

The DNA genome viruses used in this experiment were: bovine herpes virus type 4 (BHV-4) of strain FMV09-1180503; porcine circovirus 2 (PCV2b) of strain FMV05–6302 and bovine adenovirus 3 (BAV3); bovine herpes virus type 1 (BHV-1); canine parvovirus (CPV); equine herpes virus type 1 (EHV-1). The RNA genome viruses used in this experiment were: bovine viral diarrhea virus type 1 (BVDV1) of strain NADL (ATCC VR-534); swine influenza H1N1 of strain A/Swine/Saint-Hyacinthe/148/90 [59]; and Swine Influenza H3N2 of strain A/Swine/Quebec/4001/05 [60]. Cells were infected with each virus at different dilutions (1/10; 1/100; 1/1000; 1/10000; 1/1000000; 1/10000000) for 4 hours in DMEM as described for PRRSV and than treated with *AppΔapxIΔapxIIC* culture supernatant for 48 hours as described above. The infectious dose of each virus was calculated as described above for PRRSV using SJPL cells.

### Analysis of Cytokine mRNAs Expression by Real Time Reverse Transcriptase-quantitative PCR

SJPL cells and PAMs were treated and infected as described above or transfected with Polyinosinic–polycytidylic acid potassium salt (Poly (I:C)) [50 µg/mL] (Sigma-Aldrich Inc., St-Louis, USA) as a positive control for innate immunity induction, using polyethylenimine (PEI) [1 µg/µL] (Sigma) for 48 hours or treated with 1 µg/ml of lipopolysaccharide (LPS) from *E. coli* (Sigma) for 20 hours, as an IFNγ inducer. Total cellular RNA was extracted from cells using Trizol reagent (Invitrogen, Burlington, ON, Canada) according to the manufacturer’s protocol. Quantification of RNA was performed with a Nanodrop (NanoDrop Technologies, Inc., Wilmington, Delaware, USA). 1 µg of total RNA was reverse-transcribed using the QuantiTect reverse transcription kit (Qiagen, Mississauga, ON, Canada). The cDNA was amplified using the SsoFast EvaGreen Supermix kit (Bio-rad, Hercules, CA, USA). The PCR amplification program for all cDNA consisted of an enzyme activation step of 3 min at 98°C, followed by 40 cycles of a denaturing step for 2 sec at 98°C and an annealing/extension step for 5 sec at 57°C. The primers used for amplification of the different target cDNA were previously described in Provost *et al*., 2012 [19]. All primers were tested to achieve amplification efficiency between 90% and 110%. The primer sequences were all designed from the NCBI GenBank mRNA sequences using web-based software primerquest from Integrated DNA technologies. The Bio-Rad CFX-96 sequence detector apparatus was used for the cDNA amplification. The quantification of differences between the different groups was calculated using the 2^−ΔΔCt^ method. Beta-2 microglobulin (B2M) was used as the normalizing gene to compensate for potential differences in cDNA amounts. The non-infected PAMs and SJPL cells were used as the calibrator reference in the analysis.

### Statistical Analyses

A two-way ANOVA model, followed by Bonferroni post-hoc tests (Graphpad PRISM Version 5.03 software) were used to determine if a statistically significant difference exists between infections performed in adherence and cytotoxicity assays. One-way ANOVA model, followed by Tukey's Multiple Comparison Test (Graphpad PRISM) were used to determine if a statistically significant difference exists between PRRSV titer (TCID_50_) obtained in MARC-145, SJPL and PAM cells. Unpaired t tests were used for the qRT-PCR statistical analysis. Differences were considered statistically significant with a *P*<0.05.

## Supporting Information

Figure S1 **NOD1 and NOD2 inhibition effect on PRRSV replication.** Detection of the N viral protein in PRRSV infected SJPL cells by immunofluorescence. SJPL cells were infected with PRRSV MOI of 0.5 for 4 hours (B) and then treated with 100 µM of C12-iE-DAP (a NOD1 ligand) (D), or 100 µM of L18-MDP (a NOD2 ligand) (F) for 48 hours. Control are SJPL cells untreated (A) treated only with 100 µM of C12-iE-DAP (C), or 100 µM of L18-MDP (E) for 48 hours. White scale bar represents 200 µm. Pictures were taken at 100X magnification.
(TIFF)

Figure S2 **Antiviral activities of** ***AppΔapxIΔapxIIC*** **cell culture supernatant fractions against PRRSV.** Detection of the N viral protein in PRRSV infected SJPL cells by immunofluorescence. SJPL cells were untreated (A) or infected with 0.5 MOI of PRRSV for 4 hours (B) then incubated with >3 kDa (C), or <3 kDa (D), or >50 kDa (E), or <50 kDa (F) fraction of App*Δ*apxI*Δ*apxIIC cell culture supernatant. White scale bar represents 200 µm. Pictures were taken at 100X magnification.
(TIFF)
