# Supplementary figures and images for: Actinobacillus pleuropneumoniae Possesses an Antiviral Activity against Porcine Reproductive and Respiratory Syndrome Virus
Source: PLoS One. 2014 May 30;9(5):e98434. doi: 10.1371/journal.pone.0098434 (PMC4039538; doi:10.1371/journal.pone.0098434)

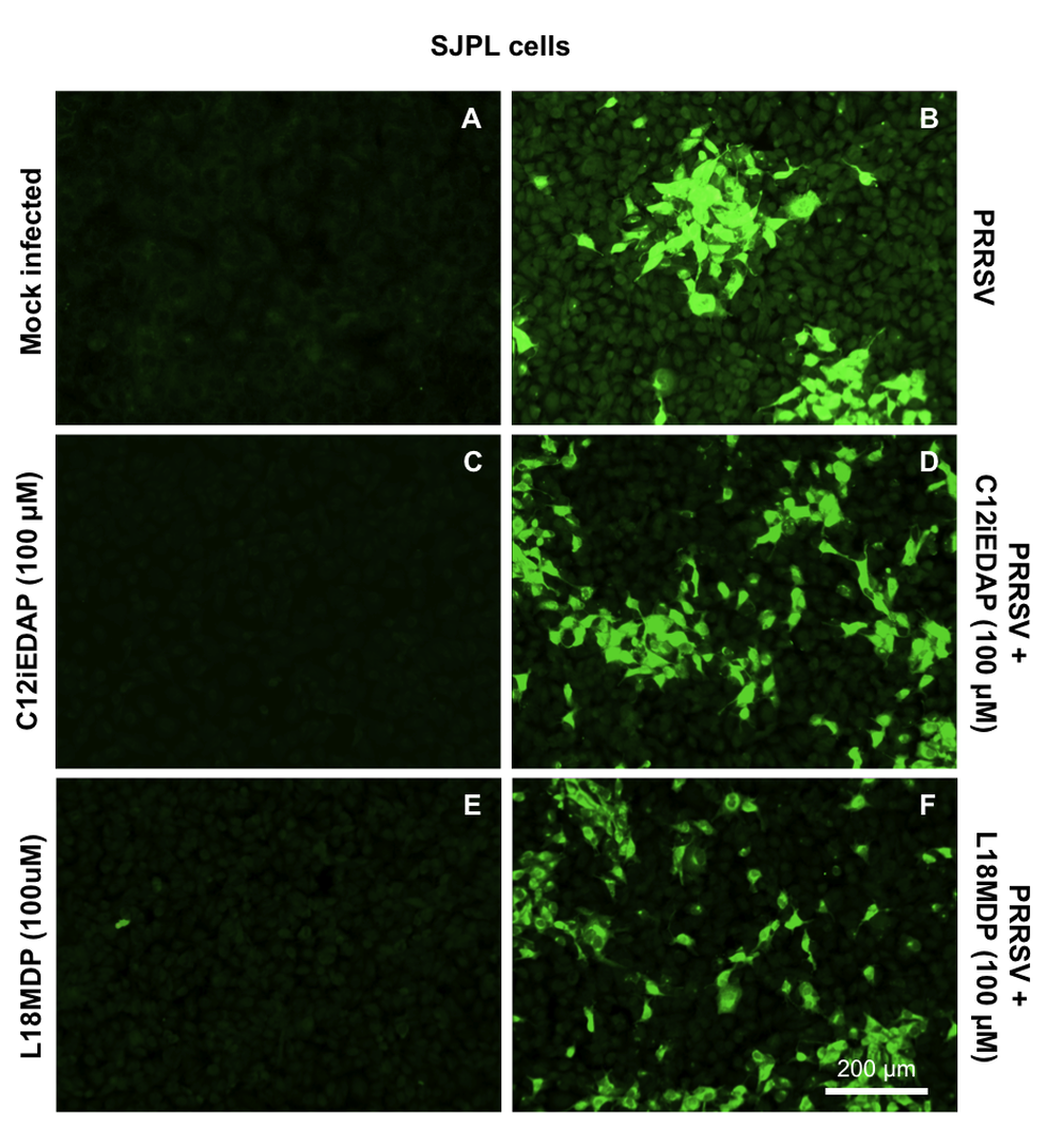

Supplement: Figure S1 — NOD1 and NOD2 inhibition effect on PRRSV replication. Detection of the N viral protein in PRRSV infected SJPL cells by immunofluorescence. SJPL cells were infected with PRRSV MOI of 0.5 for 4 hours (B) and then treated with 100 µM of C12-iE-DAP (a NOD1 ligand) (D), or 100 µM of L18-MDP (a NOD2 ligand) (F) for 48 hours. Control are SJPL cells untreated (A) treated only with 100 µM of C12-iE-DAP (C), or 100 µM of L18-MDP (E) for 48 hours. White scale bar represents 200 µm. Pictures were taken at 100X magnification. (TIFF) [file pone.0098434.s001.tiff]

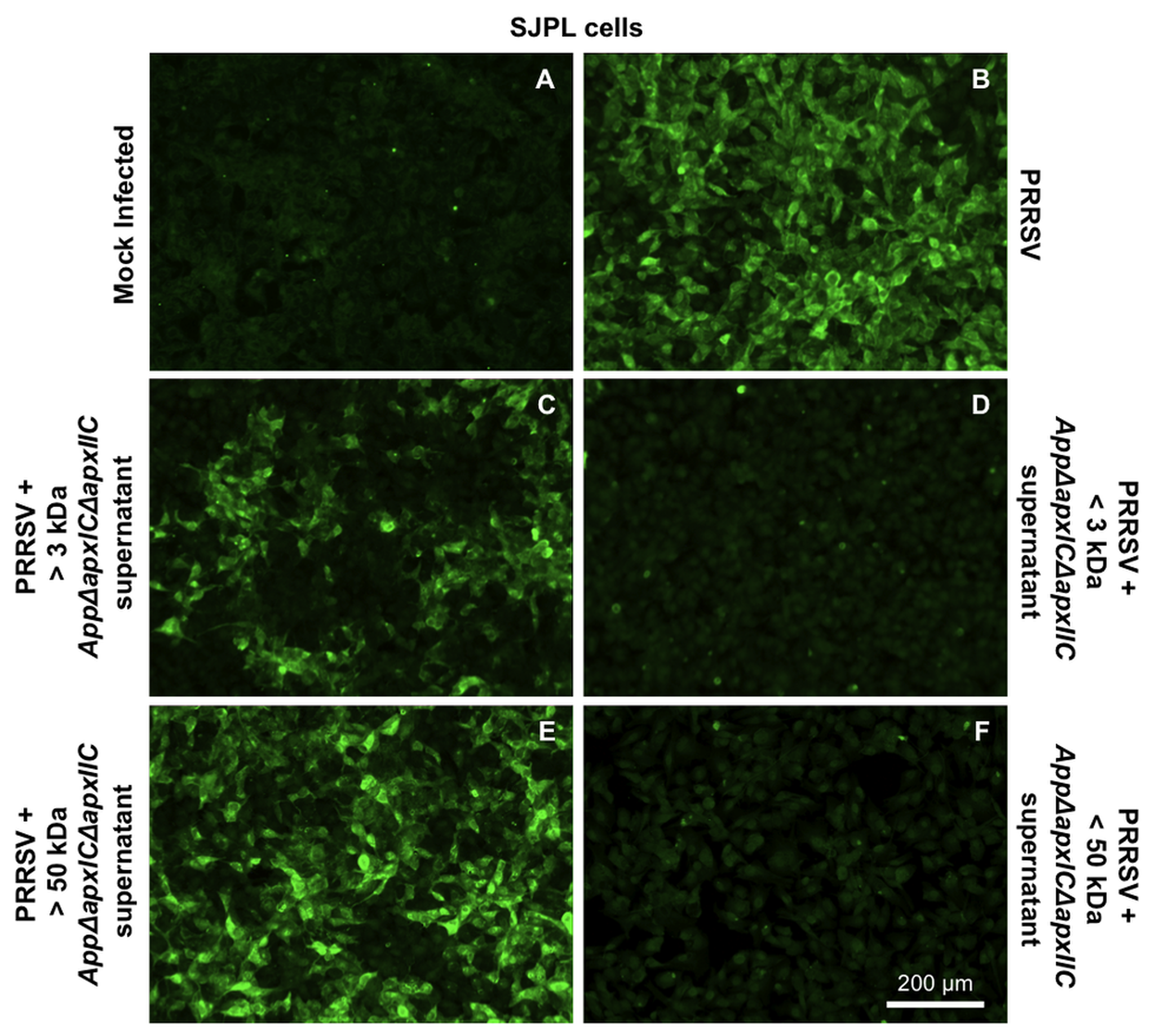

Supplement: Figure S2 — Antiviral activities of AppΔapxIΔapxIIC cell culture supernatant fractions against PRRSV. Detection of the N viral protein in PRRSV infected SJPL cells by immunofluorescence. SJPL cells were untreated (A) or infected with 0.5 MOI of PRRSV for 4 hours (B) then incubated with >3 kDa (C), or <3 kDa (D), or >50 kDa (E), or <50 kDa (F) fraction of AppΔapxIΔapxIIC cell culture supernatant. White scale bar represents 200 µm. Pictures were taken at 100X magnification. (TIFF) [file pone.0098434.s002.tiff]
